# Effectiveness of Kinesio Taping for Lymphedema in the Post-Mastectomy Patient: A Systematic Review of Randomized Controlled Trials

**DOI:** 10.3390/jcm14051700

**Published:** 2025-03-03

**Authors:** Marlena Skwiot

**Affiliations:** Faculty of Health Sciences, University of Lomza, ul. Akademicka 14, 18-400 Lomza, Poland; mskwiot@al.edu.pl; Tel.: +48-660-699-238

**Keywords:** mastectomy, lymphedema, Kinesio Taping

## Abstract

(1) **Background:** Breast cancer is one of the most common malignancies in women worldwide. Breast cancer-related lymphedema (BCRL) is a serious complication that develops as a result of damage or dysfunction of the normal functioning lymphatic system. This review aims to assess the effectiveness of Kinesio Taping in the treatment of BCRL based on randomized controlled trials. (2) **Methods:** This review was conducted in accordance with the Preferred Reporting Items for Systematic Reviews and Meta-Analyses (PRISMA) statement. Four electronic databases were searched: PubMed, Cochrane, PEDro, and Google Scholar. This study included female patients with any stage of lymphedema after mastectomy. There were no restrictions on age, race, or nationality. (3) **Results:** The literature search yielded 608 results. Eight articles met all required eligibility criteria and were included in this study. A diverse range of physical therapy interventions were used, and efficacy was measured using a variety of outcomes and measures. The summarized results indicate that Kinesio Taping applications had a positive effect on a range of outcomes, including upper limb circumference, pain, ROM, and functional status. (4) **Conclusions:** Given the modest evidence supporting the use of Kinesio Taping for the treatment of BCRL, there is a need for further prospective studies.

## 1. Introduction

Breast cancer is one of the most common malignancies in women worldwide. According to the latest statistics, breast cancer accounts for 30% of all cancer cases in women, making it the most common cancer [1].

One of the most important pillars of breast cancer treatment is surgery. In many cases, standard sparing surgery (S-BCS) is used. Another possible option is oncoplastic breast-conserving surgery (O-BCS), which involves removing the tumor in the breast and using plastic surgery techniques to reconstruct the breast, but the rate of local tumor recurrence is still debatable. In cases where breast-conserving surgery is not possible, radical mastectomy is performed [2].

Breast cancer-related lymphedema (BCRL) is a serious complication that develops as a result of damage or dysfunction of the normally functioning lymphatic system due to surgery and radiation therapy, which affects the quality of life of long-term patients [3,4]. Swelling of the extremities, pain, and limited function contribute to anxiety, depression, and other negative emotional states in patients [5].

Lymphedema can occur from a year to 30 months after surgery, which affects half of the patients. However, BCRL can develop many years after the procedure during the survival phase [6,7]. Chronic lymphedema is irreversible, difficult to treat, and significantly affects the quality of life in patients [8]. Risk factors for BCRL include young age, obesity, smoking, radical mastectomy, axillary dissection, higher level axillary lymph node dissection, comorbidities including collagenosis and hypertension, advanced stage, chemotherapy, as well as surgical complications such as postoperative bleeding and surgical site infection [9,10,11,12].

Various methods can be used to treat lymphedema after mastectomy. BCRL treatment focuses on non-pharmacological methods, including exercises, bandaging, and lymphatic drainage [13,14].

If conservative treatment is not effective enough, surgical treatment options are available. Treatments such as lymphatic bypass (LVB/A) and vascularized lymph node transplantation (VLNT) are becoming increasingly popular in reducing lymphedema. Lymphatic bypass reduces the accumulation of lymphatic fluid by anastomosing lymphatic vessels with the veins of the venous system, while VLNT enables lymphangiogenesis to improve the drainage of lymphatic fluid in the affected limb [15,16,17].

Apart from the physical effects of mastectomy, an important aspect is the psychological confidence of patients. One of the methods that have a huge impact on improving psychological parameters, including the quality of life in patients after mastectomy, is breast reconstruction [18]. Breast reconstruction methods can be divided into two main groups: methods using implants and methods using the patient’s own tissue. Fat grafts are widely used in breast reconstructive surgery. In recent years, there has been progress in techniques and improvements in procedures for the use of fat tissue in reconstructive surgery, which results in an increase in the level of satisfaction and, therefore, improvement in the mental condition of patients after mastectomy [19].

Although comprehensive therapy is considered the best technique for treating lymphedema, in clinical practice, different patient profiles are encountered, and we may need to use alternative treatment options. One of them is Kinesio Taping, which has become a new adjunctive treatment option in physiotherapy practice and has been found to be useful for body parts where compression therapy or fitting of clothing is problematic [13,14]. One of the physiological effects of Kinesio Taping is the acceleration of lympha fluctuations, causing a skin lift at the application site [20]. Studies show some efficacy of Kinesio Taping in reducing lymphedema, improving the functional status of the upper limb, reducing pain and discomfort, and improving the quality of life in patients after mastectomy with BCRL [21,22,23,24,25,26,27,28]. However, there is a limited evidence base evaluating the effectiveness of this method in treating post-mastectomy lymphedema.

This review aims to evaluate the effectiveness of Kinesio Taping in treating BCRL based on randomized controlled trials.

## 2. Materials and Methods

### Search Strategy

This review was conducted in accordance with the Preferred Reporting Items for Systematic Reviews and Meta-Analyses (PRISMA) statement and is consistent with the PRSIMA checklist. The PICO format was used in the development search strategy with search terms and limits related to mastectomy patients with lymphedema (population of interest) and Kinesio Taping (intervention).

To test the search strategy, two reviewers searched the databases independently. Search results were then compared to ensure consistency in the search process. If the results differed, the two reviewers discussed the differences together, and conflicts were resolved through discussion. A comprehensive search was developed, and four electronic databases were searched between 1 and 28 August 2024. These databases included PubMed, Cochrane, PEDro, and Google Scholar. Only English-language articles were included.

The following search terms were used as MESH headings: mastectomy, breast cancer, lymphedema, Kinesio Taping, and Kinesiology Taping.

## 3. Results

### 3.1. Study Design

RCTs were eligible for inclusion. Eligibility criteria for the population–intervention–comparator–outcome (PICO) are presented below.

#### 3.1.1. Population

This study included female patients with lymphedema after mastectomy at any stage. There were no restrictions on age, race, or nationality. Excluded were orthopedic patients, neurologic patients with lipedema, patients with edema due to circulatory diseases, patients with edema due to renal diseases, patients with edema due to thyroid diseases, and patients with edema as a reaction to drugs.

#### 3.1.2. Intervention

Because physical therapy is typically a comprehensive approach, this review was not limited to a single intervention or defined specific parameters of the intervention. However, Kinesio Taping was required to be used as a standalone intervention or in combination with other interventions.

Physiotherapy included Kinesio Taping, lymphatic drainage, bandaging, anti-edematous complex therapy, compression garments, intermittent pneumatic compression, and kinesitherapy. All interventions were conducted with the participation of a physiotherapist. Exclusion criteria included only orthopedic interventions or those using only pharmacotherapy or physiotherapy without Kinesio Taping. However, studies using complex measures (combining Kinesio Taping with other physiotherapy methods) were included.

#### 3.1.3. Comparator

The allowable comparator interventions were control (no intervention) or usual care.

#### 3.1.4. Outcome

Given the multidimensional nature of BCRL-related complaints, such as discomfort, pain, limited range of motion, and functional impairment of the upper limb, the search was not limited to any specific outcomes. Outcomes of interest included pain, upper limb functional status, and quality of life.

### 3.2. Studies Selection

All the literature search results were transferred to the EndNote X9 reference management software to sort identified studies. Duplicate results were removed with EndNote X9. Studies were then accepted or removed by checking the title and abstract against PICO criteria. The full texts of eligible studies were analyzed to determine their eligibility for PICO criteria.

### 3.3. Methodolical Quality

Included studies were assessed and ranked according to the ‘intervention categories’ of the National Health and Medical Research Council (NHMRC) evidence hierarchy. The Modified McMaster Critical Review Form for quantitative studies was used to assess the methodological quality of the included studies in accordance with the guidelines. This tool assessed eight main elements: study purpose; literature review; study design (all experimental designs); sample (description of participants, justification for size, ethics, and consent); outcomes (reliability and validity, outcome measures, and measures used); intervention (description, contamination, and interaction); results (statistical and clinical significance, methods of analysis, and waivers); and conclusions with implications for practice (limitations and biases). Individual elements were scored as ‘yes’, ‘no’, ‘not addressed’, or ‘NA not applicable’. A score of ‘1’ was given for ‘yes’ and ‘0’ for ‘no and not addressed’; if the category “NA” was used, the total score was changed accordingly. Depending on the study design and relevant items, the maximum total score for a study could be fourteen.

### 3.4. Synthesis of Results

Due to the heterogeneity of the included studies, a meta-analysis was not performed. Instead, a descriptive synthesis was undertaken.

**Figure 1:**
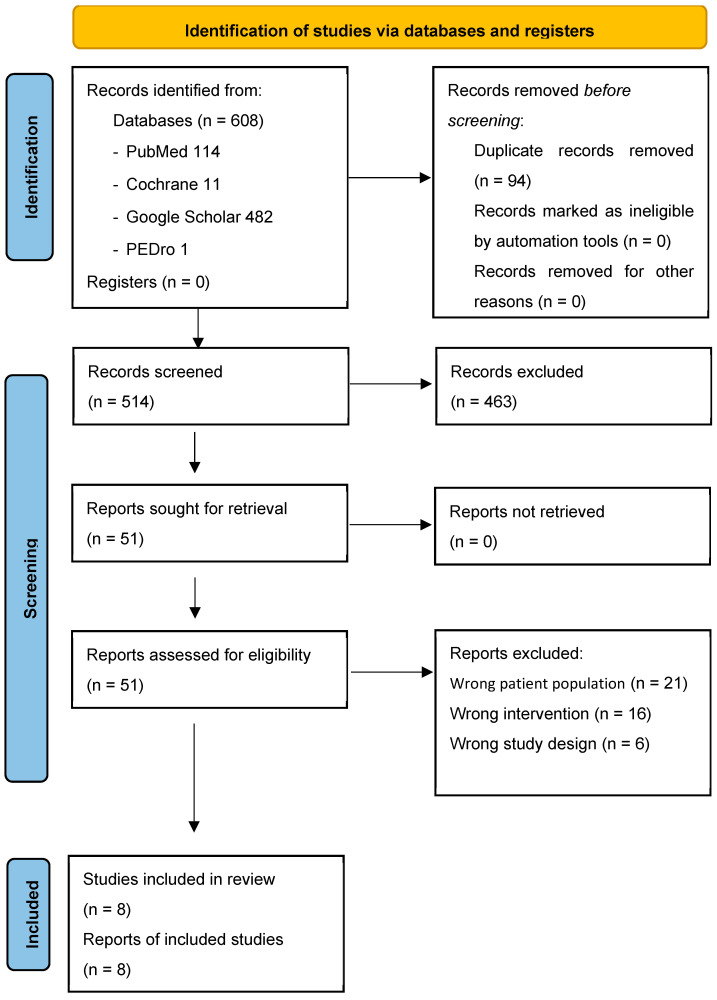
PRISMA 2020 flow diagram.

The literature search yielded 608 results, of which 94 duplicates were removed. After screening 514 titles and abstracts, 463 items were removed. A total of 51 articles were fully analyzed, of which 41 were excluded: 21 because the study population did not meet the inclusion criteria, 6 for inappropriate study design, and 16 for inappropriate intervention. Eight articles met all the required eligibility criteria and were included in the study. The PRISMA flowchart is presented in Figure 1.

### 3.5. Risk of Bias

Table 1 provides an overview of the NHMRC levels of evidence and assigned scores based on the Modified McMaster Critical Review Form for the eight eligible studies. All study designs were randomized controlled trials.

### 3.6. Study Characteristics

Table 2 summarizes the study characteristics. This systematic review identified different study designs, but all of them were randomized controlled trials. Studies were conducted in Turkey (x2), Egypt (x2), Poland (x2), and Spain (x2). They were published between 2013 and 2023. These studies examined the effectiveness of physiotherapy interventions using Kinesio Taping applications on reducing pain, improving upper limb function, reducing lymphedema, and improving quality of life in patients with BCRL.

### 3.7. Participants’ Characteristics

A total of 226 post-mastectomy patients participated in the studies. The number of participants ranged from 15 to 45, with an age of M = 58.9 years. The average BMI value was 28.7; one study [26] reported that seven people were obese (BMI > 30 kg/m^2^). The stage of lymphedema was identified in all studies, including six studies reporting patients with stages II and III [22,24,25,26,27,28], while the remaining two reported patients with stage II only [21,23]. The mean time after surgery was 30.2 months (min. 7.6 ± 1.7 [28]; max. 59.7 ± 127.3 [21]).

### 3.8. Intervention Type

Although all studies examined the use of physiotherapy in the treatment of upper limb lymphedema, there was notable variability in both the interventions used and the manner in which they were applied. One study used a single intervention (Kinesio Taping) [22], while the remaining studies used more than one intervention, making it difficult to determine causality. Interventions included bandaging [21,27,28], exercise [21,23,28] pneumatic compression [25,26], compression garments [24], and MLD [25,26,27].

The similarities in the application of Kinesio Taping tapes were the use of a fork shape, i.e., lymphatic applications, and the direction from distal to proximal. However, variations in tape tension ranging from 0% to 15–20% have been reported. Applications were changed from 1 to 3 times a week. In all studies, interventions were delivered by a physiotherapist.

The exercises used focused primarily on improving ROM and strengthening the upper limb [21,23,28]. They consisted of squeezing the hands using a ball and active resistance exercises of the shoulder, elbow, and wrist joints using a resistance band [21], as well as isometric exercises and stretching [23].

### 3.9. Outcome Measures (OMs)

The types of outcome measures used to assess the effectiveness of physiotherapy interventions using Kinesio Taping varied across studies; however, all of them measured upper limb biometry (circumferences). For this purpose, differences in volume and circumference were measured. Both subjective and objective measures were used to check the effectiveness of therapy in the remaining domains: pain (PDQ, SPADI, Likert, and VAS), range of motion (goniometer), upper limb functionality (QuickDASH), handgrip strength (dynamometer), and quality of life (LYM-QoL ARM, EORTC QLQ-C30).

Studies varied in time points; outcomes were measured from 10 days to 12 weeks. One study did not report the duration of the study [28]. No studies reported adverse events due to physiotherapy intervention. The range of domains and outcome measures used in each study are given in Table 3.

#### 3.9.1. Functional Status of the Upper Extremity

- Limb circumference

The primary goal of physiotherapy interventions in the eligible studies was to improve the functional status of the upper limb, including reducing lymphedema. In eight studies, the circumferences of the upper limb undergoing therapy were measured. Various tools were used to assess the size of the lymphedema before and after therapy. To assess the limb circumference, the following tests were performed: measurements at four points, metacarpal phalangeal joints, wrists, 10 cm distal to the lateral epicondyle, and 12 cm proximal lateral epicondyles. Patient’s position for examination: sitting on a chair with shoulders at a 90-degree angle [21,23,24]. Circumferences were also measured in the prone position, with elbows straight and arms relaxed at the sides. They were measured starting from 3 cm at the styloid process of the elbow and ending at 45 cm proximally and at the metacarpal and middle palm bones [22]. Yet another tool for assessing limb volume was the optoelectronic Perometer 40 T, connected to a personal computer [26]. In five studies [21,22,23,24,27], a reduction in circumferences was noted after the end of the intervention. In three studies [25,26,28], the intervention did not produce a similar effect. It should be noted that additional interventions and the duration of therapy differed in individual studies. Namely, in one study, the effect was observed as early as 10 days after the start of the intervention, and the therapy also included MDL, exercises, and bandaging [27]. Two studies observed a significant change after 4 weeks with additional exercises [23] and compression garments [24] and after 4 and 12 weeks also using exercises and bandaging as interventions [21]. Improvement was also found 3 weeks after the intervention using only Kinesio Taping [22].

- ROM

To assess the functional status of the patients, the ROM of the upper limb was measured in three studies [21,24,25]. A goniometer was used in all experiments. Physiotherapy had a positive effect on improving painless range of motion in the upper limb in two of the three studies [21,24].

- General functioning

In two studies [21,23], the general functioning of the patients was assessed using the QuickDASH scale. Improvement was noted in both studies—after 12 weeks and after 4 weeks from the start of the intervention. It should be emphasized that in both studies, which confirmed a statistically significant improvement in general functioning, exercises were an additional intervention.

- Grip strength

Three studies [22,23,25] examined the grip strength of patients before and after physiotherapy intervention using a dynamometer. Two studies found improvement in this parameter after the intervention.

#### 3.9.2. Pain

Although pain was not the main reason for physiotherapy intervention, five out of eight studies measured the reduction in symptoms using a subjective pain scale: VAS [27,28], PDQ [21], SPADI [22], and Likert [24]. Four studies reported a positive effect of physiotherapy interventions on pain reduction at various times after the start of the intervention. In the study by Torres et al., no improvement was noted.

#### 3.9.3. Quality of Life

Two studies [21,22] examined the quality of life in patients using the EORTC QLQ-C30 and LYM-QoL ARM questionnaires, respectively. The intervention consisted of Kinesio Taping alone [22] and in combination with bandaging [21], and a positive effect on quality of life was obtained after 3 and 12 weeks.

### 3.10. Summary of Results

Table 4 summarizes the results of all eight studies in the individual domains. A beneficial effect of the interventions was found in all domains studied. The results indicate a reduction in pain and an improvement in upper limb function. The results obtained in the eight studies are encouraging, especially in terms of a reduction in upper limb circumference, an increase in the range of motion, and a reduction in pain. Despite the promising effects, caution is required when interpreting the results due to the small number of studies.

### 3.11. NHMRC FORM Framework

Table 5 presents a synthesis of the results using the NHMRC FORM framework. The study results are favorable and encouraging, but the differences in the interventions provided and the small number of studies lowered the overall recommendation. Although these results may be helpful in BCRL physiotherapy, caution should be exercised in implementing these recommendations.

## 4. Discussion

This systematic review aimed to address the evidence base for physiotherapy interventions using Kinesio Taping in post-mastectomy patients with lymphedema. There was a modest evidence base consisting of eight randomized controlled trials. The summarized results indicate that Kinesio Taping may have a positive effect on several domains, such as upper limb functional status, upper limb biometry, ROM, pain, and quality of life. Consistent evidence for the effectiveness of physiotherapy was seen primarily for pain, general function, and quality of life, suggesting that physiotherapy interventions may be effective in improving the quality of life in patients. Despite these positive findings, caution should be exercised in interpreting the results due to quantitative limitations and the lack of uniformity in the evidence base.

The use of physiotherapy interventions in patients with BCRL identified positive effects in several domains using both subjective and objective measures. This highlighted the potential role of Kinesio Taping in this specific group of patients.

In the accompanying evidence, intervention times varied significantly (from 10 days to 12 weeks). This may have a significant impact on the results obtained. In a review on the effectiveness of MLD in reducing lymphedema, it was reported that the duration of the intervention (1 month) and the age of patients under 60 years of age were important factors of effectiveness [29].

There is evidence to support the effectiveness of other alternative interventions in reducing lymphedema in post-mastectomy patients, including low-level laser therapy and endermology [30]. There were reports confirming the effectiveness of low-level laser therapy in reducing upper limb volume compared to the control group [31,32,33]. When analyzing the effectiveness of Kinesio Taping in upper limb biometry, there were insufficient data in the endermology group. No significant differences were found in the reduction in arm circumference between the intervention group and the control group [34,35,36]. No changes in the pain scale were found when comparing the endermology group with the control group.

E.H. Rannikko et al. [37] conducted the first-ever randomized prospective clinical trial to quantitatively demonstrate a positive effect of gene therapy on lymphedema, although there were no differences between groups in primary outcome measures. Various functional tests have been used to assess the effectiveness of physiotherapy interventions in patients with BCRL. Due to its ease of administration, simplicity, and repeatability, the QuickDASH [38,39] is a commonly used tool. Using this scale, two studies in this review [21,22] demonstrated that Kinesio Taping improved upper limb function and patient comfort.

Evidence was found of a reduction in the circumference of the upper limb with lymphedema after surgical treatment [40,41,42]. There were differences between studies in preoperative conservative intervention protocols, but all patients were treated with compression garments postoperatively, regardless of the type of procedure. Postoperative improvement in patients’ quality of life was noted, both in the physical sphere and in the improvement of mood, using LYMQOL [43,44].

Preliminary studies on the long-term prevention of BCRL have shown high effectiveness using new surgical methods, such as the Simplified Lymphatic Microsurgical Preventing Healing Approach (SLYMPHA), which enables the immediate reconstruction of the lymphatic system. New surgical methods are playing an increasingly important role in the prevention of BCRL [45].

One of the problems with the effective prevention and treatment of BCRL is the current lack of standardized assessment tools that would enable researchers to compare their results. In this review, several different assessment methods were used to assess the extent of edema, which may produce inconsistent results. In order to advance the effectiveness of lymphedema interventions, attention should be paid to patient education and the systematization of diagnostic tools and clinical techniques of researchers [46].

Peres C. et al. [47] measured the degree of impairment, topography, and biophysical changes in subcutaneous lymphedema tissue following breast cancer treatment using ultrasonography. The results showed a significant difference in echogenicity and thickness between the affected and healthy upper limb, throughout the entire upper limb.

For patients after mastectomy, pain and limited ROM of the upper limb associated with the scar and BCRL itself constitute significant problems. A systematic review by Lin Y. et al. [48] provided evidence on the reduction in these ailments with the use of aerobic and resistance exercises. Effects were found on reducing pain intensity, improving shoulder flexion and internal rotation range, reducing upper limb dysfunction, improving muscle strength in arm flexion and abduction, and reducing the incidence of arm lymphedema. De Sire A. et al. [49] emphasized the importance of comprehensiveness in the context of the rehabilitation of patients with BCRL, by integrating a specialized home-based exercise program with specialized treatment and self-care training.

Studies have shown a significant negative impact of BCRL on quality of life, including physical, psychological, and social domains [50]. Two studies reported improved mood after the completion of physiotherapy [21,22], using standardized questionnaires LYM-QoL ARM and EORTC QLQ-C30. This is likely due to the positive results of reduced pain and improved upper limb functionality.

People with BCRL who have persistent lymphedema after the completion of primary treatment report significantly poorer quality of life than those without edema. There is an advantage in assessing the impairment caused by lymphedema using appropriate, multidimensional quality of life assessment tools. It is reasonable to seek strategies for the prevention and treatment of BCRL to minimize its impact on physical, psychological, and social well-being across the cancer continuum [50].

## 5. Limitations

Although this work is based on best practices for conducting systematic reviews (PRISMA), it has limitations. This review was conducted using electronic databases, implementing gray literature and secondary search strategies, which are complex and imprecise compared to the black literature. As a result, some studies may have been unidentified and omitted. Another limitation was the exclusion of non-English-language publications.

Eight publications were ultimately identified that met the inclusion criteria, representing a modest body of evidence, although with consistent positive findings. Therefore, the generalization of the results to the BCRL patient population is not warranted.

## 6. Conclusions

Given the modest evidence supporting the use of Kinesio Taping physiotherapy interventions in reducing BCRL, there is a need for further prospective studies. More randomized controlled trials, with large samples, controlled groups, and interventions with long-term follow-up, could help identify the strong effect of Kinesio Taping on improving patient health in both physical and psychological domains.

## 7. Practical Implications

There is evidence to support the use of Kinesio Taping in patients with BCRL. The positive impact of the intervention was noted in outcomes across several domains, including pain, ROM, upper extremity biometrics, and functional status. Kinesio Taping in combination with other methods may increase the effectiveness of interventions in the treatment of lymphedema. It may also be considered as an alternative treatment option for lymphedema, especially in patients who tolerate conservative methods less well. However, due to the small evidence base, caution should be exercised when implementing these recommendations.

## Figures and Tables

**Table 1:** An overview of the NHMRC levels of evidence and assigned scores based on the Modified McMaster Critical Review Form.

	NHMRC Level	Items on Modified Mcmaster Critical Review Form																Raw Score	
Study		1	2	3	4a	4b	4c	5a	5b	6a	6b	6c	7a	7b	7c	7d	8		%
**Yilmaz** [21]	Level II	Y	Y	Randomized Controlled Trial	15	Y	N/A	Y	Y	Y	Y	No	Y	Y	Y	Y	Y	12 out of 13	92%
**Basoglu** [23]	Level II	Y	Y	Randomized Controlled Trial	20	Y	N/A	Y	Y	Y	Y	No	Y	Y	Y	No	Y	11 out of 13	85%
**Otero** [24]	Level II	Y	Y	Randomized Controlled Trial	15	Y	N/A	Y	Y	Y	Y	No	Y	Y	Y	No	Y	11 out of 13	85%
**Taradaj** [25]	Level II	Y	Y	Randomized Controlled Trial	45	Y	N/A	Y	Y	Y	Y	No	Y	Y	Y	No	Y	11 out of 13	85%
**Tantawy** [22]	Level II	Y	Y	Randomized Controlled Trial	30	Y	N/A	Y	Y	Y	Y	Y	Y	Y	Y	Y	Y	13 out of 13	100%
**Pekyavaş** [27]	Level II	Y	Y	Randomized Controlled Trial	30	Y	N/A	Y	Y	Y	Y	No	Y	Y	Y	No	Y	11 out of 13	85%
**Torres** [28]	Level II	Y	Y	Randomized Controlled Trial	29	Y	N/A	Y	Y	Y	Y	No	Y	Y	Y	Y	Y	12 out of 13	92%
**Smykla** [26]	Level II	Y	Y	Randomized Controlled Trial	42	Y	N/A	Y	Y	Y	Y	No	Y	Y	Y	Y	Y	12 out of 13	92%
**NAD: not addressed; N/A: not applicable**					226														

**Table 2:** The study characteristics.

Study	Country	Sample Size	Mean Age	Gender	Injury	Symptoms	Co-Intervention	Outcome Addressed	Follow Up
**Yilmaz** [21]	Turkey, 2023	15	51.4 (±10.7)	F: 15	Mastectomy	Lymphedema II grade	Bandaging, exercise	Measurement of upper limb circumference, shoulder joint ROM, and Q-DASH	12 weeks
**Basoglu** [23]	Turkey, 2021	20	53.7 ± 8.6	F: 20	Mastectomy	Lymphedema II grade	Exercise	Upper limb biometry (circumference and volume), grip strength, and Q-DASH	4 weeks
**Otero** [24]	Spain, 2019	15	N/A	F: 15	Mastectomy	Lymphedema II and III grade	Compression garment		4 weeks
**Taradaj** [25]	Poland, 2016	G 1: 22 G2: 23	G 2: 63.2 ± 5.1; G 2: 63.2 ± 5.1	F: 45	Mastectomy	Lymphedema II and III grade	G1: KT, with MLD and intermittent pneumatic compression; G2: combined with intermittent pneumatic compression and MLD	Volume difference (mL), grip strength, and ROM	N/A
**Tantawy** [22]	Egypt, 2019	30	54.3 ± 4.16	F: 30	Mastectomy	Lymphedema II and III grade		Limb circumference, SPADI, Handgrip strength (dynamometer), and EORTC QLQ-C30	3 weeks
**Pekyavaş** [27]	Turkey, 2014	G1: 15 G2: 15	56.5 ± 9.4, 58 ± 8.5	F: 30	Mastectomy	Lymphedema II and III grade	MLD, exercise program, and bandaging	Lymphedema volume, VAS	10 days
**Torres** [28]	Spain, 2020	29	59.6 ± 10.6	F: 29	Mastectomy	Lymphedema II and III grade	Bandaging	Lymphedema volume, VAS	N/A
**Smykla** [26]	Poland, 2013	G1: 20, G2: 22	G1: 67.34, G2: 65.43	F: 42	Mastectomy	Lymphedema II and III grade	MLD, pneumatic compression	Lymphedema volume	1 month

N/A: not applicable.

**Table 3:** Outcome domain and outcome measures used in each study.

Study	Outcome Domain and Outcome Measures									
	Pain				Functional Status of the Upper Extremity				QOL	
	PDQ	SPADI	Likert	VAS	ROM	Quick DASH	Limb Circumference	Handgrip Strength (Dynamometer)	LYM-QoL ARM	EORTC QLQ-C30
					(Goniometer)					
**Yilmaz** [21]	✓				✓	✓	✓		✓	
**Basoglu** [23]						✓	✓	✓		
**Otero** [24]			✓		✓		✓			
**Taradaj** [25]					✓		✓	✓		
**Tantawy** [22]		✓					✓	✓		✓
**Pekyavaş** [27]				✓			✓			
**Torres** [28]				✓			✓			
**Smykla** [26]							✓			

Legend: PDQ—pain detect questionnaire; ROM—range of motion; SPADI—shoulder pain and disability index; VAS—visual analog scale; QOL—quality of life; LYM-QoL ARM—Lymphedema Quality of Life Tool; EORTC QLQ-C30—European Organization for the Research and Treatment of Cancer Quality of Life Questionnaire.

**Table 4:** The results of all eight studies in the individual domains.

Study	Effects of Physiotherapy Interventions									
	Pain				Functional Status of the Upper Extremity				QOL	
	PDQ	SPADI	Likert	VAS	ROM	Quick DASH	Limb Circumference	Handgrip Strength (Dynamometer)	LYM-QoL ARM	EORTC QLQ-C30
					(Goniometer)					
**Yilmaz** [21]	(+) ↓				(+) ↑	(+) ↑	(+) ↓		(+) ↑	
**Basoglu** [23]						(+) ↑	(+) ↓	(+) ↑		
**Otero** [24]			(+) ↓		(+) ↑		(+) ↓			
**Taradaj** [25]					(=)		(=)	(=)		
**Tantawy** [22]		(+) ↓					(+) ↓	(+) ↑		(+) ↑
**Pekyavaş** [27]				(+) ↓			(+) ↓			
**Torres** [28]				(=)			(=)			
**Smykla** [26]							(=)			

Legend: PDQ—pain detect questionnaire; ROM—range of motion; SPADI—shoulder pain and disability index; VAS—visual analog scale; QOL—quality of life; LYM-QoL ARM—Lymphedema Quality of Life Tool; EORTC QLQ-C30—European Organization for the Research and Treatment of Cancer Quality of Life Questionnaire; (+)—improvement; (=)—without changes; ↓—reduction; ↑—increase.

**Table 5:** NHMRS FORM framework.

Component	Grade	Comments
**Evidence base**	B—Good One or two level II studies with a low risk of bias	Quantity: 8 studies Participants: 226 post-mastectomy patients with lymphedema Level II: 8 studies Level III-2: 0 studies Level III-3: 0 studies Level IV: 0 studies
**Consistency**	C—Satisfactory Some inconsistency reflecting genuine uncertainty around clinical questions	Some inconsistency reflecting genuine uncertainty around clinical questions Findings consistent Multiple study designs Heterogeneous interventions Varied outcome measures and time point measurements
**Clinical impact**	B—Substantial	Consistent findings for outcomes: in particular functional status of the upper extremity All studies have statistical significance The clinical significance should be approached with caution No adverse effects reported
**Generalizability**	B—Good Population/s studied in the body of evidence are similar to the target population for the guideline	Population of studies is similar to the target Age of M = 58.9 years All patients had grade II or III upper limb lymphedema (post-mastectomy) Studies conducted in four different countries that have different health care contexts
**Grade of recommendations**	C—Satisfactory Evidence provides some support for recommendation(s), but care should be taken in its application	These studies had high evidence and were of moderate methodological quality Although overall there were positive results, the current evidence base is not homogeneous in terms of interventions delivered and parameters and results measured for post-mastectomy patients

## Abbreviations

The following abbreviations are used in this manuscript:

OMs	Outcomes measures
BCRL	Breast cancer-related lymphedema
PDQ	pain detect questionnaire
ROM	range of motion
SPADI	shoulder pain and disability index
VAS	visual analog scale
QOL	quality of life
LYM-QoL ARM	Lymphedema Quality of Life Tool
EORTC QLQ-C30	European Organization for the Research and Treatment of Cancer Quality of Life Questionnaire
